# Validation of the English version of the Autism-Spectrum Quotient in an English-speaking Singaporean sample

**DOI:** 10.1371/journal.pone.0291726

**Published:** 2023-09-28

**Authors:** Rachael Tan, Chris Ashwin

**Affiliations:** Centre for Applied Autism Research, Department of Psychology, University of Bath, Bath, United Kingdom; University of Valencia: Universitat de Valencia, SPAIN

## Abstract

The Autism-Spectrum Quotient (AQ) measures the degree of autistic traits in clinical and non-clinical samples and has been validated in various countries and languages. However, the AQ has not been validated in Singapore, an Asian country whose population speaks predominantly English. Although previous validation studies have examined the distribution of scores, internal consistency, test-retest reliability and construct, convergent and discriminant validities in Asian countries using translated versions of the AQ and generally shown a suitable structure of the AQ, other studies testing cultural differences of the AQ have provided inconsistent results about whether differences exist in scores between Western and Asian samples. Additionally, while prior literature has consistently documented sex differences in AQ scores, findings about the relationship between personality traits and friendship quality with autistic traits have been mixed. The aim of the current study was to validate the psychometric properties of the original English AQ in a non-clinical Singaporean sample and compare their mean AQ scores to previous Western samples. In this study, psychometric properties of the original English AQ were assessed in 113 Singaporean adults (47M/66F; Mean age = 37.78; SD = 14.52) with no clinical diagnoses. They completed the AQ, the Friendship Questionnaire (FQ) and the short Big Five Inventory, with a subsample completing the AQ twice within three to six months. Results showed that AQ scores were normally distributed and the AQ had satisfactory internal consistency and test-retest reliability and it demonstrated construct, convergent and discriminant validities. Higher AQ scores were related to lower friendship quality and extraversion and higher neuroticism. The mean AQ scores of the Singaporean sample did not differ to that reported in original British sample. Together, present findings showed the original English AQ to be reliable for measuring the degree of autistic traits in a non-clinical Singaporean sample, producing comparable AQ scores and showing the same relationships to other social and personality measures and the same sex differences as has been reported in English samples. This supports the use of the AQ in Singapore for clinical and research purposes and suggests that the measurement of autistic traits in some Asian cultures is comparable to that reported in Western cultures.

## 1. Introduction

Autism spectrum disorders (ASD) are characterized by difficulties in social communication and interaction along with restricted interests and repetitive behaviours [1]. Autistic traits are also seen to some degree in people without an ASD diagnosis, which includes relatives of individuals with an ASD diagnosis, referred to as the broader autism phenotype (BAP) [2]. For example, people demonstrating the BAP are reported to show poorer turn-taking and difficulties with shifting conversational topics and coping with change in a similar way to those with a diagnosis of ASD [3].

Various questionnaires have been developed to measure autistic traits in both clinical and non-clinical samples, including the Social Responsiveness Scale (SRS) [4] and the Autism-Spectrum Quotient (AQ) [5]. Studies using these measures have reported that autistic traits exist on a spectrum ranging from low degrees in non-clinical individuals to higher degrees in individuals diagnosed with ASD and they are normally distributed in the general population [6, 7]. Most autism literature to date has focused on its clinical presentation, with disproportionately less research on autistic traits in non-clinical individuals without an ASD diagnoses to help further our understanding of the wider autism spectrum [8].

### 1.1 Validation of the AQ

The AQ was designed as a screening tool to indicate the likelihood of ASD and has become the most widely used measure of autistic traits in non-clinical populations [9]. The original 50-item AQ is a self-report questionnaire that assesses the degree of autistic traits and produces a total score from zero to 50 [5]. The AQ is freely available and quick and easy to administer for in-person or online research. The AQ was initially validated in a non-clinical British sample comprising university students and adults who were not current students, and results showed moderate to high internal consistency (i.e. Cronbach’s alpha coefficients), a continuous distribution of AQ scores which indicated a spectrum of autistic traits, higher AQ scores in males than females which demonstrated construct validity, and good test-retest reliability [5]. These satisfactory psychometric properties were found in both the student and adult samples despite differences in socioeconomic status, educational level and intelligence quotient. This suggested that AQ scores were unaffected by these factors, increasing the generalisability of its reliability and validity. The homogeneity within the sample of students with degrees ranging from sciences to humanities and adults with diverse occupations and the large age range of the sample further strengthen the validity of the AQ throughout the non-clinical population. Given the strengths of the AQ, studies assessing similar psychometric properties of the AQ in non-clinical samples have consistently shown the original English AQ to be validated in other English-speaking Western countries, such as Australia and the United States of America [10, 11].

The AQ has also been validated in non-clinical samples in Asian countries including Japan, Korea and China, although they have generally used translated versions instead of the original English version [12–14]. The AQ has been translated into many different languages including Japanese, Korean, Chinese etc., to facilitate the selection of representative samples not limited to proficient English speakers and reduce possible confounding by differences in English proficiency. For example, Wakabayashi and colleagues [13] successfully replicated the results from Baron-Cohen and colleagues’ study. [5] using the Japanese version of the AQ and a non-clinical Japanese sample with results demonstrating the Japanese AQ had a continuous score distribution, good internal consistency, test-retest and inter-rater reliabilities, and good construct validity. Findings from these studies in Asian sample have generally suggested the AQ is suitable for measuring autistic traits across both Western and Asian cultures.

However, other studies have reported cultural differences in the scoring and responses on the AQ between in Asian compared to Western samples, although results to date have been inconsistent to each other. For example, Freeth et al. [15] found moderate to good internal consistency of the 50 item AQ across Malaysian, Indian and British student samples, although the Malaysian and Indian students had higher AQ scores compared to the British students which they interpreted as showing cultural differences in the reporting or interpretation of autistic traits in other countries to where the AQ was developed. A study by Chee et al., [16] tested cultural differences using the 28-item abridged version of the AQ [17] in Malaysian and Dutch samples, with the Malaysian sample choosing either the English or the Malaysian language version of the AQ to complete. Results showed a suitable and stable structure of the AQ in both the Dutch and Malaysian samples, however the Malaysian sample had significantly higher AQ scores compared to the Dutch sample which once again was interpreted to suggest cultural differences in the interpretation or reporting of autistic traits. Another study carried out in Malaysia also reported that Mandarin speaking Malaysian students scored higher in the English version of the AQ than the Mandarin version, but further reported that Bahasa speaking Malaysians scored the same across both the Bahasa and English versions of the AQ [18]. Together, studies have generally shown a suitable and stable structure of the AQ in Asian samples, but with mixed findings about whether participants score higher in the English versus native Asian language versions of the AQ. However, the evidence to date has mainly involved student samples, has not generally controlled for mental health or language proficiency, and has focused mainly on Malaysian samples within Asia. More convincing evidence about cultural differences in AQ responses would be tested in samples from the general population all completing the same English version of the AQ and controlling for mental health diagnoses, in an Asian country apart from Malaysia with high English language proficiency.

Additionally, convergent validity of the AQ has been demonstrated by its relationship with other measures that assess related underlying constructs, including friendship quality and the Big Five personality traits. The AQ and friendship quality assess the same construct of social functioning [15, 16]. Correspondingly, Sedgewick and colleagues [21] examined the relationship of the AQ with the unidimensional relationship closeness scale (URCS) in a non-clinical sample and found a negative correlation between scores on the AQ and URCS, demonstrating that higher autistic traits relate to reduced friendship quality. However, the URCS design excludes participants who cannot identify any close friends. It is likely that selection bias occurred when those likely to have higher AQ scores were excluded, which limits the generalisability of results, elucidating a gap in current research. Alternatively, the use of a dimensional measure ranging from no close friends to a high level of friendship quality is more accessible and would provide stronger validation results that are generalisable throughout the non-clinical population, regardless of the presence of close friendships. As for the Big Five personality traits, studies in non-clinical British and Japanese samples have also reported a negative relationship between AQ scores and extraversion and a positive relationship between AQ scores and neuroticism [18, 19]. These studies have also demonstrated discriminant validity with AQ scores not being correlated to the other traits of conscientiousness, openness and agreeableness given that these constructs are not theoretically related to autistic traits [20]. However, only two studies have reported these effects and the studies included exclusively Western samples, necessitating replication in an English-speaking Asian sample to further theoretical understanding on the relationship between autistic and personality traits and possible cross-cultural differences.

### 1.2 Rationale

Critically, no published reports to date have validated any standardised measures of autistic traits in Singapore, so the main aim of this study was to validate the AQ in a non-clinical Singaporean sample and compare the findings with AQ validation studies with Western samples. Singapore is an interesting country because its located in Asia but English is predominantly spoken, presenting a unique opportunity to administer the original English version of the AQ to a non-clinical Asian sample without compromising the selection of a representative sample, unlike many previous Asian studies. This establishes a common basis for comparison with previous Western studies that similarly used the original English version of the AQ. Since ASD diagnoses are less socially accepted in Singapore compared to many Western countries and may be associated with under-diagnosis and underreporting of prevalence rates [21], validation of the AQ in Singapore can help facilitate research on autistic traits in Singaporean adults and more effective screening of ASD.

### 1.3 The present study

The present study aimed to validate the original English version of the AQ in a non-clinical Singaporean sample by testing its internal consistency, test-retest reliability and nature of distribution of scores. Additionally, it aimed to test construct validity by assessing sex differences in autistic traits and convergent and discriminant validities by examining the relationships between friendship quality and personality traits with autistic traits, and to compare mean AQ scores in the Singaporean sample with those from the British sample in the original English AQ validation study. These psychometric properties and analyses were chosen for their precedence in prior Western and Asian AQ validation studies to serve as a basis for comparison to validate the AQ in this new non-clinical sample with similar criteria and enable accurate assessment of the cultural independence of the AQ and autistic traits [5, 10–14]. The hypotheses were: (1) the AQ will have satisfactory internal consistency and test-retest reliability, (2) AQ scores will be normally distributed, (3) males will score significantly higher than females on the AQ to demonstrate construct validity, (4) AQ scores will be significantly negatively correlated to FQ scores and extraversion and significantly positively correlated to neuroticism to demonstrate convergent validity, and (5) AQ scores will not be significantly correlated to openness, conscientiousness and agreeableness to demonstrate discriminant validity. We did not have a set hypothesis about whether the AQ mean score in the Singaporean sample would be different to the mean AQ score in the original British sample given the mixed results to date about AQ scores in Asian versus Western samples.

## 2. Methods

### 2.1 Participants

A total of 181 participants (*M*_age_ = 37.78, *SD* = 14.52; 62Male/ 100Female/ 19 not indicating sex) were recruited in Singapore using social media platforms (e.g., Facebook, Instagram etc.) and adverts posted in social locations. The inclusion criteria included citizenship or permanent residency in Singapore, speaking fluent English, being 18 years or older, and not reporting any clinical diagnoses. 39 participants had responses below 20% completion, indicating that they did not complete all items on the AQ, thus, were excluded. 25 participants had AQ scores higher than the clinical cut-off of 26 [22] and four participants reported having a clinical diagnosis (e.g. depression) and so were excluded for being clinically relevant. This created a final sample of 113 participants (*M*_age_ = 38.35, *SD* = 14.63; 47M/ 66F) who all indicated a sex whose data were included in the analysis.

### 2.2 Materials

#### 2.2.1 Autism-Spectrum questionnaire [5]

The AQ is a 50-item self-administered questionnaire measuring the degree of autistic traits in adults comprising five subscales, each with ten items: Social Skills, Attention Switching, Attention to Detail, Communication and Imagination. An example item is “I find social situations easy.”. Responses were measured on a 4-point Likert scale from “Definitely Disagree” to “Definitely Agree” and given a score of 1 for answers indicating the presence of an autistic trait and 0 for the absence of an autistic trait. Total scores ranged from 0 to 50 with higher scores indicating higher degrees of autistic traits. The AQ has been validated in clinical and non-clinical Asian and Western samples [5, 12, 13]. Baron-Cohen and colleagues [5] reported satisfactory internal consistency with moderate to high Cronbach’s alpha coefficients for each subscale (Social Skills = .77, Attention Switching = .67, Attention to Detail = .63, Communication = .65, Imagination = .67).

#### 2.2.2 Big Five Inventory–Short (BFI-S) [23]

The BFI-S is a 15-item self-administered questionnaire comprising five subscales, each with three items: Neuroticism, Extraversion, Openness to Experience, Agreeableness and Conscientiousness. An example item is “I see myself as someone who is talkative”. Responses were measured on a 5-point Likert scale from “Disagree Strongly” to “Agree Strongly”. Scores are totalled for each subscale and each has a range from 0 to 15. The BFI-S has been validated in terms of convergent and discriminant validities and Cronbach’s alpha coefficients for each subscale were moderate (Neuroticism = .60, Extraversion = .66, Openness to Experience = .63, Agreeableness = .50, Conscientiousness = .60) [24].

#### 2.2.3 Friendship Questionnaire (FQ) [25]

The FQ is a 35-item self-report questionnaire which measures the quality of friendships, with an example item being “How easy do you find discussing your feelings with your friends?” Total scores range from zero to 135 with higher scores indicating higher quality friendships. The FQ is reported to have a high Cronbach’s alpha coefficient of .84 in a non-clinical sample [21].

### 2.3 Procedure

The study received ethical approval from the Psychology Research Ethics Committee (PREC) in the Department of Psychology at the University of Bath, and all participants provided informed written consent. The study was completed online using Qualtrics to present the survey and to record responses, and the order of the survey was the demographic questions, the AQ, the BFI-S and the FQ. To examine test-retest reliability, 41 participants completed the AQ twice, with the second completion being 3 to 6 months after their first completion.

### 2.4 Statistical design

To validate the continuous distribution of AQ scores and determine the nature of the data, normality of AQ scores was tested using the Kolmogorov-Smirnov test. The results indicated a normal distribution in the non-clinical Singaporean sample, *D*(113) = .06, *p* = .200.

Internal consistency for AQ total score was calculated using Cronbach’s alpha. Test-retest reliability was determined by comparing the difference in scores of the first and second AQ attempts using a paired-samples t-test. To test if the AQ was culture independent, an independent samples t-test was conducted on the mean AQ scores of the Singaporean sample in this study and of the British sample from Baron-Cohen and colleagues’ [5] study. To test construct validity, another independent samples t-test was done on the mean AQ scores of female and male participants. Cohen’s *d* was used to calculate effect size. Lastly, to test convergent and discriminant validities, two multiple linear hierarchical regressions were conducted with AQ scores as the DV. One regression included age and sex as model 1 and personality traits as Model 2, while the other had age and sex as Model 1 and friendship quality as Model 2.

## 3. Results

### 3.1 Internal consistency

The results from the reliability analysis showed that the internal consistency of the total AQ was satisfactory and Cronbach’s alpha coefficient was moderate (α = .64).

### 3.2 Culture independence of the AQ

The mean AQ score of the Singaporean sample (*M* = 15.43, *SD* = 4.86) was not significantly different to the mean score reported in the British sample (*M* = 16.40, *SD* = 6.30) from the original AQ study by Baron-Cohen and colleagues [5], *t*(112) = -0.20, *p* = .844.

### 3.3 Construct validity

The mean AQ score of males (*M* = 16.72, *SD* = 4.49) was significantly higher than that of females (*M* = 14.52, *SD* = 4.94), *t*(111) = 2.43, *p* = .017, indicating a significant sex difference.

### 3.4 Convergent validity

The results from the hierarchical linear regression with AQ score as the DV showed that that Model 1 significantly predicted AQ score, *R*^*2*^ = .06, *F*(2, 110) = 3.51, *p* = 0.033. Sex was the only variable that significantly predicted AQ score (see Table 1). Results showed that Model 2 also significantly predicted AQ score, *R*^*2*^ = .32, *F*(7, 105) = 6.92, *p* < .001, and that *R*^*2*^ values for Model 2 were significantly greater than Model 1, *R*^2^ = .26, Δ*F*(5, 105) = 7.84, *p* < .001, indicating that it explained significantly more of the variance (see Table 1). In Model 2, only extraversion and neuroticism significantly predicted AQ score, with extraversion showing a negative relationship and neuroticism showing a positive relationship.

**Table 1:** Regression coefficients of age, sex and personality traits on AQ score.

Variable	*B*	*SE B*	β	*p*	*R*	*R* ^*2*^
Model 1						
Constant	20.49	2.11			.25	.06
Age	-0.03	0.03	-.10	.293		
Sex	-2.39	0.93	-.24	.011		
Model 2						
Constant	21.11	3.82			.56	.32
Age	-0.01	0.03	-.02	.857		
Sex	-2.55	0.82	-.26	.002		
Conscientiousness	0.21	0.17	.11	.218		
Agreeableness	-0.16	0.19	-.08	.386		
Openness	-0.17	0.18	-.09	.369		
Extraversion	-0.56	0.14	-.36	< .001		
Neuroticism	0.59	0.15	.37	< .001		

The results from the second hierarchical linear regression with AQ score as the DV showed that Model 1 significantly predicted AQ score, *R*^*2*^ = .06, *F*(2, 110) = 3.51, *p* = 0.033. (see Table 2). Results further showed that Model 2 significantly predicted AQ scores, *R*^*2*^ = .11, *F*(3, 104) = 4.28, *p* = .007, and that the *R*^*2*^ value for Model 2 was significantly greater than Model 1, Δ*R*^2^ = .04, Δ*F*(1, 104) = 4.30, *p* = .041. Regarding individual predictors, friendship quality was found to significantly and negatively predict AQ scores.

**Table 2:** Regression coefficients of age, sex and friendship quality on AQ score.

Variable	*B*	*SE B*	β	*p*	*R*	*R* ^*2*^
Model 1						
Constant	21.24	2.17			.27	.07
Age	-0.04	0.03	-.12	.211		
Sex	-2.60	0.95	-.26	.007		
Model 2						
Constant	25.96	3.12			.33	.11
Age	-0.05	0.03	-.15	.116		
Sex	-2.24	0.96	-.22	.021		
Friendship quality	-0.06	0.03	-.20	.041		

### 3.5 Test-retest reliability

Results showed that AQ scores for the first (*M* = 15.85, *SD* = 5.23) and second completions (*M* = 16.39, *SD* = 6.63) were not significantly different to each other, *t*(40) = -1.07, *p* = .290, and were strongly correlated, *r*(40) = .88, *p* < .001, indicating satisfactory test-retest reliability. The mean scores of participants who did (*M* = 16.10, *SD* = 5.03) and did not (*M* = 15.06, *SD* = 4.76) attempt the AQ again were not found to be significantly different, *t*(111) = -1.10, *p* = .275, showing that the sample who did the test-retest reliability was comparable to the sample that did not complete it.

## 4. Discussion

### 4.1 Findings

This is the first study to validate a standardized measure of autistic traits in Singapore, which was demonstrated using the original English version of the AQ. The results showed that the AQ had satisfactory internal consistency, good test-retest reliability, demonstrated construct validity based on males scoring higher than females, and that AQ scores were normally distributed. Convergent validity was demonstrated with negative relationships found between AQ scores and scores for the FQ and extraversion along with a positive relationship between AQ scores and neuroticism, while discriminant validity was shown by the lack of significant correlations between AQ scores with scores on the other Big 5 personality traits of openness, conscientiousness and agreeableness. Lastly, the mean AQ score in the current Singaporean sample was not significantly different to the mean AQ score of the original British non-clinical sample, showing further validation of the English AQ. Together, the results supported all hypotheses and revealed the AQ to be a reliable and valid measure of autistic traits in a non-clinical Singaporean sample, comparable to prior studies which have used Western samples. The findings support the use of the English AQ in Singapore to screen for ASD and assess the degree of autistic traits in research studies and, potentially, in other Asian countries with English-speaking participants. The findings also suggest that autistic traits are reported and interpreted in a comparable way across Singaporean and British cultures when the same language is used for the measurement of autistic traits.

The internal consistency and test-retest reliability reported in this study were satisfactory and within the range of previous validation studies, supporting the first hypothesis [5, 10–14]. While previous studies demonstrated the culture independence of the AQ by validating translated versions, this is the first study to fully validate the original English version in an Asian sample. This study provides stronger and direct evidence that the AQ is robust to cultural differences in social norms and cultural dimensions, such as individualism and collectivism, which are associated with Western and Asian cultures respectively. The present findings suggest that the original English AQ version can be reliably used with English-speaking Asian populations and further strengthens theoretical understanding of the cultural independence of autistic traits. These facilitate further potential cross-cultural application of autism literature and clinical and support practices.

The AQ scores were normally distributed in the non-clinical Singaporean sample, supporting the second hypothesis and demonstrating consistency with previous studies of autistic traits in non-clinical samples in both Western and Asian countries [4, 5, 14]. This finding verifies the presence of autistic traits in non-clinical individuals and indicates that autistic traits vary in degrees across general population samples. It supports the concept of a spectrum of traits in the general population, providing evidence for this in an Asian culture using the original English AQ version. This concept updates the traditional understanding of autism as a disorder distinct from the general population to the idea that autistic traits are present to some degree amongst most individuals in the population, including both males and females [6]. The current finding of higher AQ scores in male than female participants supports the third hypothesis and is consistent with the extensive literature showing sex differences in autistic traits [4, 5, 26, 27]. Their difference in scores support the empathising-systemising and extreme male brain theories proposing that males typically show more characteristics of ASD than females, often demonstrated as higher systemising abilities and lower empathising abilities, and that an extreme version of this profile is characteristic of individuals diagnosed with ASD [28]. The present results reveal that the same sex differences in autistic traits are evident in a non-clinical Asian sample when assessed with the original English AQ, showing comparable effects in both Western and Asian cultures when controlling for potential translation issues.

The negative correlation between AQ and FQ scores provided convergent validity for the AQ, showing that increased autistic traits in non-clinical Singaporean individuals related to lower friendship quality which supports the first part of the fourth hypothesis. This is consistent with previous findings using various measures of autistic traits and friendship quality in clinical and non-clinical Western samples [3, 21]. Non-clinical individuals with high autistic traits likely experience some social difficulties, such as determining appropriate self-disclosure and providing emotional support, which compromise their ability to form close friendships [29]. Additionally, exhibiting restricted and repetitive interests and behaviours may involve fixation on certain conversational topics and activities. This may interfere with group dynamics and lead others to form unfavourable judgements about them, likely resulting in others’ reduced intentions to initiate and pursue social interactions with them [30]. Thus, higher autistic traits in non-clinical individuals may affect their social skills and efforts to develop close friendships and lead to lower quality friendships [15, 31]. As their underlying constructs have been theoretically established to be related, the significant correlation found between AQ and FQ scores validates the English version of the AQ for use in Singaporean samples.

The relationship found between autistic traits and extraversion and neuroticism in the Singaporean sample are consistent with previous studies relating the AQ and Big Five personality traits, supporting the second part of the fourth hypothesis [21, 22]. Non-clinical individuals with lower autistic traits are more likely to be adept at navigating social situations, to find social interactions rewarding and to actively pursue them compared to those with higher autistic traits, leading to the positive relationship between autistic traits and extraversion [32]. The former may also experience negative affect less frequently during social interactions and in response to unexpected changes in routines and circumstances, resulting in the positive relationship between autistic traits and neuroticism [33]. Additionally, the lack of significant correlations between openness, conscientiousness and agreeableness with autistic traits indicates that the AQ can reliably discriminate between measuring autistic traits and these personality traits, supporting the fifth hypothesis. Collectively, the relationships between different personality traits and autistic traits validates the AQ and extends research and understanding of the personality correlates of autistic traits in Asian cultures.

Finally, the mean AQ score of the non-clinical Singaporean sample was not significantly different compared to the mean AQ score of the non-clinical British sample reported in the original AQ study [5], once again showing validity of the English version of the AQ in the Singaporean sample because the overall degree of autistic traits was comparable to those reported in Western countries. This finding is consistent with a recent study that reported a Malaysian sample scored the same on the Bahasa Malaysian version of the AQ as on the original English version of the AQ [18], showing comparable scoring across both the English and Malaysian versions of the AQ. In the same study another sample, who were Mandarin speaking Malaysians, showed greater scores in the English AQ compared to the Mandarin AQ. However, the Mandarin Malaysian sample had reduced English proficiency compared to their proficiency in Malaysian, with the language differences interpreted as contributing to the AQ score differences. Since the current study recruited people using English language adverts which stipulated fluency in English language was needed to participate, its likely the current sample also had high English language proficiency similar to the Bahasa Malaysian sample. Together, these findings demonstrate that when language proficiency is controlled, comparable scores are found across English and Asian versions of the AQ, suggesting that the degree of autistic traits are recognised and reported in similar ways across both Western and Asian cultures.

The validation of the AQ in Singapore, evident from the results supporting all the hypotheses, provides important clinical screening and research purposes for the country. National Institute for Health and Care Excellence guidelines in the United Kingdom recommend the use of the AQ as an initial step for adults without moderate or severe learning disabilities who suspect that they have ASD to identify if they need a comprehensive clinical assessment [34]. Currently, adults in Singapore who suspect that they have ASD generally start with a clinical appointment involving interviews with family members, but social stigma often deters this step and subsequent access to support resources and services [35]. The AQ provides a quick and easily administered alternative step ahead of clinical appointments to streamline ASD assessment and diagnosis in Singapore, increasing the availability of and speed of accessing support. The validation of the AQ also has research implications for testing the relationship between autistic traits and other constructs in Singaporean samples, critically adding to autism literature currently disproportionately based on Western samples [36]. Together, these may help raise public awareness about the autistic spectrum in Singapore towards reduced stigma and greater acceptance [37].

### 4.2 Limitations

Limitations of the study include that the sample was relatively small for a validation study, due to a number of participants not completing all the measures and our conservative threshold for determining clinically relevant AQ scores. This meant we could also not perform a factor analysis on the data in order to test the factor structure in the Singaporean sample compared to other Western samples. This may also account for the relatively lower, although still acceptable level, of internal consistency (α = .64) reported in this study relative to previous studies [5, 13, 14]. However, the AQ scores reported here were still consistent with those reported in other Western studies and numerous different measures which demonstrated reliability and validity of the English AQ in the Singaporean sample, providing validity to the present findings. Further studies can test larger samples to investigate the factor structure of the AQ in Singapore and compare the factors to those reported in other Western and Asian samples.

In conclusion, the findings here demonstrated satisfactory internal consistency, good test-retest reliability, a normal distribution of scores, and construct, convergent and discriminative validity of the English AQ in the Singaporean sample, along with mean AQ scores that were not different to the original British sample. Together, the present results validated the AQ as a reliable measure of autistic traits in a non-clinical Singaporean sample, with both the AQ and autistic traits shown to be robust to cultural differences when compared to the same original English version. This supports the use of the AQ as a screening tool to indicate the likelihood of ASD in adults for clinical purposes and allows for new avenues of research testing the degree of autistic traits in Singaporean samples and potentially other English-speaking Asian samples.

## Supporting information

S1 Data (CSV)
